# Systemic influences of dietary NAD^+^ supplementation relevant to the search for ocular hypertensive therapies for glaucoma

**DOI:** 10.1038/s41598-026-58123-8

**Published:** 2026-07-29

**Authors:** Kacie J. Meyer, Adam Hedberg-Buenz, Kai Wang, Michael G. Anderson

**Affiliations:** 1 Department of Molecular Physiology and Biophysics, University of Iowa, 375 Newton Road, Iowa City, IA 52242 USA; 2 Institute for Vision Research, University of Iowa, Iowa City, IA USA; 3 Department of Biostatistics, University of Iowa, Iowa City, IA USA; 4 Department of Ophthalmology and Visual Sciences, University of Iowa, Iowa City, IA USA

**Keywords:** Diseases, Medical research, Neuroscience

## Abstract

Dietary nicotinamide (NAM) is neuroprotective in rodent models of glaucoma. With dietary delivery, additional systemic influences of NAM are possible, prompting us to test for influences on non-neuronal phenotypes relevant to glaucoma. Test cohorts of mice on high-fat diets differed by NAM content (control, “CON-diet”; 2,000 mg/kg/day supplementation, “NAM-diet”) and by ocular treatments inducing various levels of ocular hypertension (Naïve; Ad5-Empty; Ad5-ATF4). Masked observers assessed intraocular pressure (IOP), body weight, and slit-lamp appearance. Naïve and Ad5-Empty cohorts were analyzed for gut microbiome composition. Regardless of diet, Naïve cohorts maintained consistent IOP. On CON-diet, the Ad5-ATF4 and Ad5-Empty cohorts developed IOP elevation. Based on results from a linear mixed-effects model evaluating the effects of diet and ocular treatment on IOP, NAM-diet lowered IOP in only the Ad5-Empty cohort. NAM-diet also prevented weight gain associated with a high-fat diet. NAM-diet was linked to altered alpha and beta diversity of the gut microbiome, including changes to a butyrate-producing genus, *Faecalibaculum*. Among the systemic influences resulting from dietary NAM consumption, NAM’s ability to affect both neuroprotection and IOP would be favorable for glaucoma therapy. The possibility of dietary NAM influencing body weight and microbiome composition in humans warrants additional investigation.

**Supplementary Information:**

The online version contains supplementary material available at https://doi.org/10.1038/s41598-026-58123-8.

## Introduction

Reduced nicotinamide adenine dinucleotide (NAD^+^) levels impair mitochondrial efficiency and contribute to age-related disease. NAD^+ ^is a critical coenzyme and co-substrate for cellular metabolism^1^. As a coenzyme, NAD^+ ^and its phosphorylated derivative (NADP) act as hydride acceptors or donors (NADH, NADPH). They interconvert during intermediary reactions without being consumed and support cellular energy demands that otherwise may become depleted with aging^2–4^. As a co-substrate, NAD^+ ^is essential for multiple enzyme classes that strongly influence aging^5,6^. Increased NAD^+^ levels can protect retinal ganglion cells in both ex vivo models^7–9^ and various in vivo animal models of glaucoma^10–14^. Accordingly, elevating NAD^+ ^is a proposed therapy for age-related diseases^15,16^, including glaucoma^17,18^.

Cellular NAD⁺ is often boosted via systemic supplementation with precursors like nicotinamide (NAM), nicotinamide riboside (NR), or nicotinamide mononucleotide (NMN). A key initial glaucoma study used DBA/2J mice treated with 2,000 mg/kg/day NAM in water and high-fat chow^10^. Similarly, systemic administration of NR has been used in mouse studies^11–13^ and human clinical trials^18^. Dietary NAM supplementation is neuroprotective^10^, and an oral therapy for glaucoma could have advantages for medication compliance. However, it is also non-selective in delivery and could have additional non-neuronal influences. In the eye, it is particularly important to consider the hypothesis that dietary NAM might influence glaucomatous neurodegeneration through a mechanism of lowering intraocular pressure (IOP)—the main risk factor for glaucoma. Systemic NAM supplementation could theoretically also have broad effects. These may be especially pronounced in the gastrointestinal tract, where NAM concentrations would be particularly high following consumption.

This study investigated how systemic NAD^+ ^supplementation via a NAM-rich diet affects IOP, body weight, and gut microbiome composition. To align with previous work in mice, we compared 2,000 mg/kg/day NAM-enriched versus control high-fat diets^10^. Three treatment groups were studied: (1) the “Naïve” group, consisting of unmanipulated C57BL/6J (B6) mice used to assess NAM’s effect on IOP in healthy animals; (2) the “Ad5-ATF4” group, consisting of B6 mice with bilateral intravitreal injections of replication-deficient adenovirus serotype 5 carrying the *activating transcription factor 4* (*ATF4*) gene, which induces ocular hypertension in mice^19^; and (3) the “Ad5-Empty” group, consisting of B6 mice with bilateral intravitreal injections of vehicle control. Over 7 weeks, masked observers assessed IOP, body weight, and slit-lamp appearance. Fecal samples from Naïve and Ad5-Empty mice were collected at week 6 to evaluate microbiome composition. These data were used to refine conditions under which NAM might affect IOP and to explore related changes in body weight and the gut microbiome.

## Results

### Influence of dietary NAM on IOP

Following baseline IOP measurements and assignment of mice into matched groups differing by diet (CON-diet or NAM-diet) and treatment (both eyes: Naïve, Ad5-Empty injections, or Ad5-ATF4 injections), IOP, slit-lamp appearance, and body weight were assessed by masked observers over a 7-week period. Left and right eye IOPs were significantly correlated at baseline (*p* < 0.0001; Supplemental Materials 1 and 2). Naïve mice maintained this correlation over time (*p* = 0.005), but Ad5-Empty and Ad5-ATF4 injected mice did not (*p* = 0.711 and *p* = 0.658, respectively). Because the values were not independent at baseline, IOP was averaged from the left and right eye measurements per mouse per time point in subsequent analyses.

**Fig. 1:**
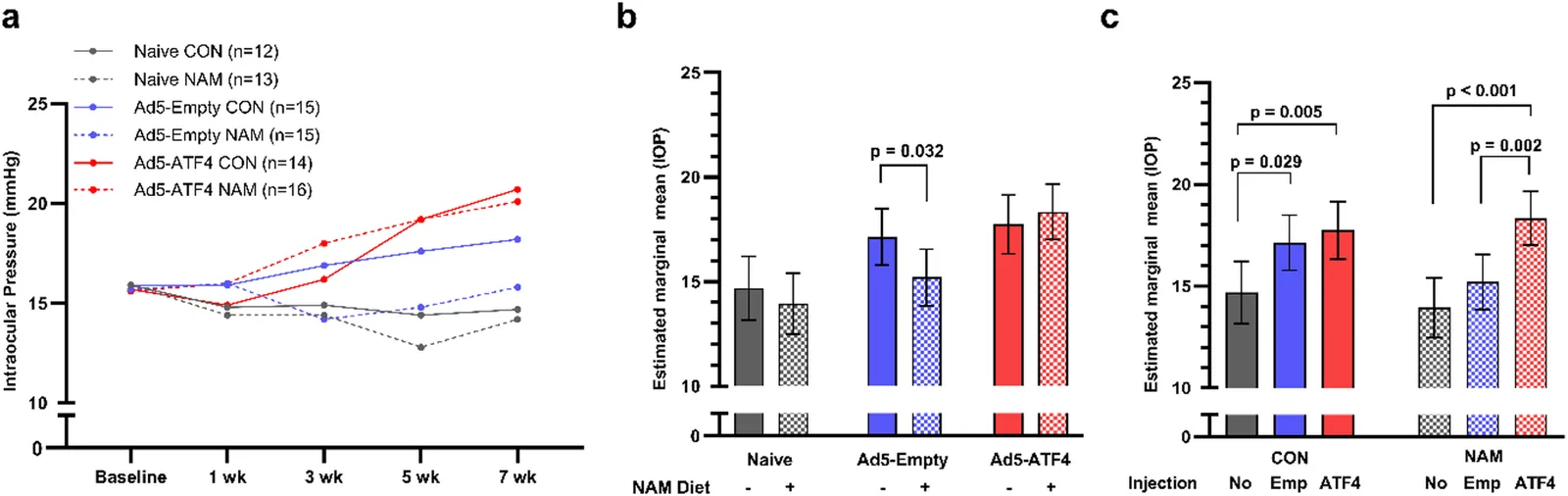
Effect of NAM-diet on IOP. **(a)** Mean IOP from cohorts of C57BL/6J mice differing in ocular treatment and diet, measured by rebound tonometry over time. Note, the cohorts had matched IOP by design at baseline. **(b)** EMMs of IOP, calculated from the 3-, 5-, and 7-week time points, by diet within each injection group with 95% confidence interval. **(c)** EMMs of IOP, calculated from the 3-, 5-, and 7-week time points, by injection within each diet group with 95% confidence interval. CON = CON-diet, NAM = NAM-diet, n = number of mice (IOP values of both eyes were averaged to yield one IOP metric per animal), IOP = intraocular pressure, No = no injection (i.e., Naïve), Emp = Ad5-Empty, ATF4 = Ad5-ATF4, p = EMMs pairwise comparisons with Tukey test.

As expected for groups that were selected to initially have matched averages, IOP was similar between groups at baseline but, over time, the influences of the ocular treatments became increasingly evident (Fig. 1A). Based on analysis with a linear mixed model (LMM), there was not an overall significant effect of NAM as a fixed effect (*p* = 0.948). Estimated marginal means (EMMs) analysis for diet within each injection group indicated that NAM had no significant overall effect on the IOP of Naïve or Ad5-ATF4 subgroups but did affect the Ad5-Empty subgroup (Fig. 1B). EMMs analysis for injection within each diet group indicated that on the CON diet, injection had an overall significant influence on IOP for the Ad5-ATF4 and Ad5-Empty subgroups in comparison to the Naïve subgroup, but they did not differ statistically in comparison to one another (Fig. 1C).

### Influences on ocular health

**Fig. 2:**
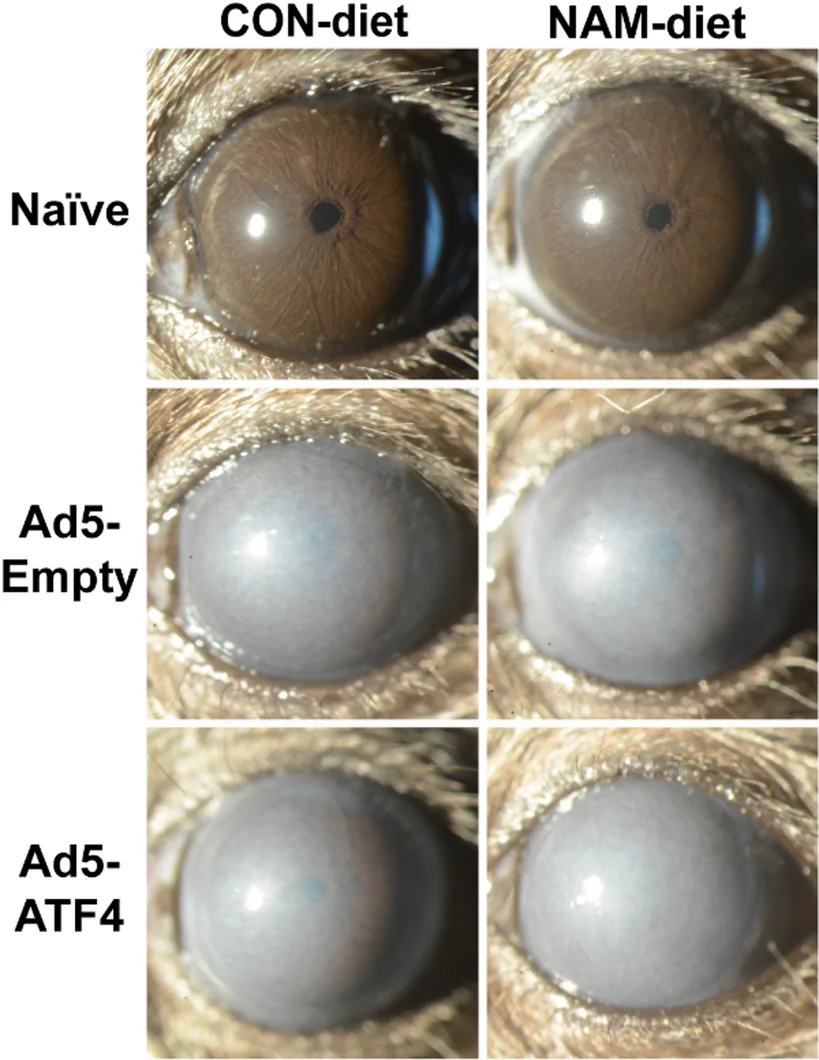
Representative images showing anterior segment slit-lamp images with broad beam illumination. All images from the 7-week time point.

All eyes were examined by slit-lamp. No eyes showed signs of needle-related complications. As expected, there was a mix of eyes with either wild-type appearances or of those injected with Ad5 (Fig. 2)^20^. All Naïve eyes appeared normal (50 of 50 eyes). In Ad5-injected mice, 89 of 120 corneas were cloudy in all exams, and another 19 were intermittently cloudy. In 10 eyes, only a “dusty” iris appearance was noted, likely due to mobilized macrophages. Both eyes of a single Ad5-injected mouse had no discernable visible abnormalities. Slit-lamp appearances did not differ between Ad5-Empty and Ad5-ATF4 cohorts.

### IOP and body weight correlation

**Fig. 3:**
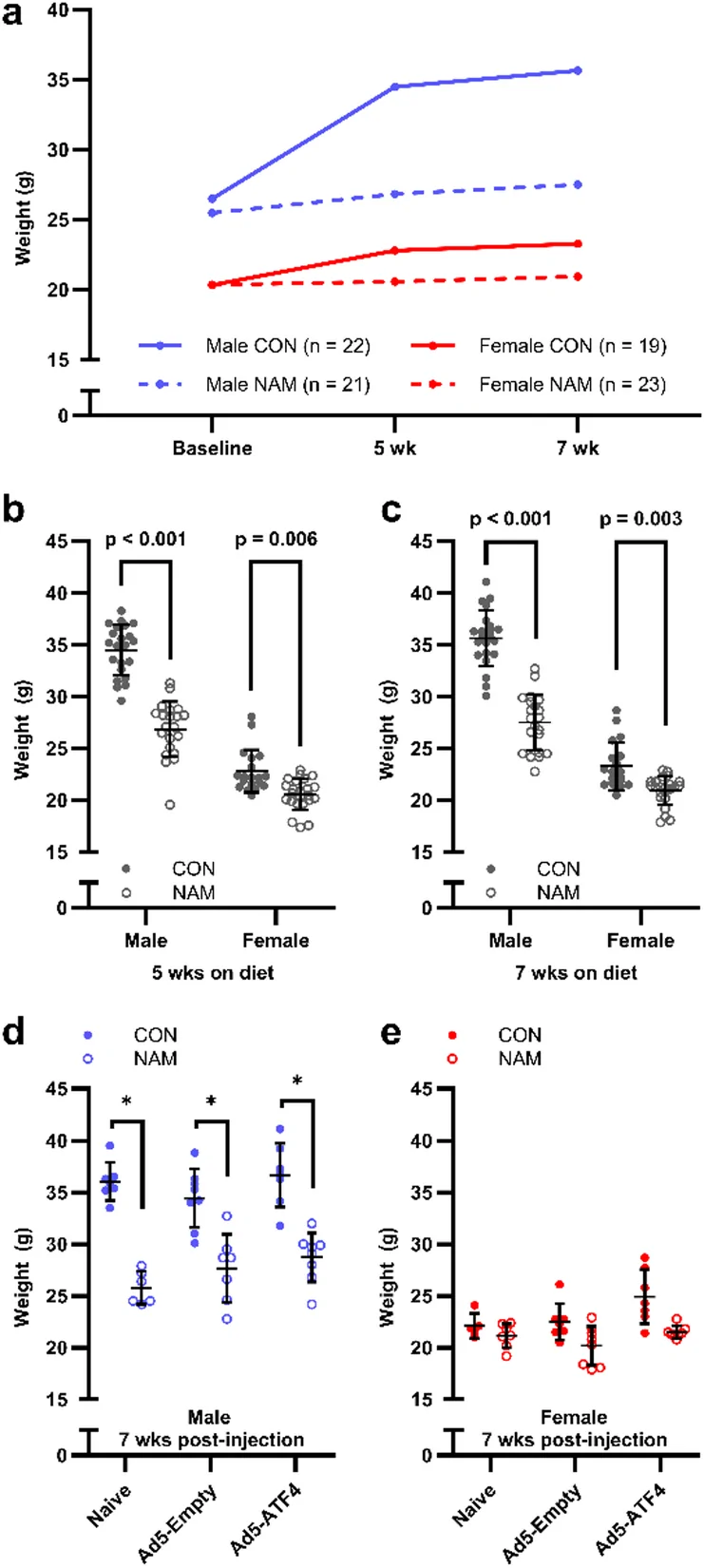
Effect of NAM-diet on body weight. (**a**) Mean body weight from cohorts of C57BL/6J mice differing in diet over time. Both diets were high fat. (**b**,** c**) A subset of the same data are replotted showing statistical comparisons of male and female mice on CON- and NAM-diets at 5- and 7-weeks. (**d**,** e**) A subset of the same data are replotted showing statistical comparisons of injection groups on CON- and NAM-diets at 7-weeks for male and female mice. Each point represents one mouse, with mean ± standard deviation for each group. CON = CON-diet, NAM = NAM-diet, n = number of mice, p = EMMs pairwise comparisons with Tukey test, * = *p* < 0.001.

Both diets were high fat, matching prior work by Williams et al.^10^. As expected, body weight increased, especially in males (Fig. 3A)^21^. Based on analysis with a LMM, NAM attenuated this gain (Fig. 3B and C)^22^, with statistically significant responses detected in both sexes at the 5-week (male *p* < 0.0001; female *p* = 0.001; EMM pairwise comparisons with Tukey) and 7-week (male *p* < 0.0001; female *p* < 0.0001) time points. Ocular treatment had no effect on weight (Fig. 3D and E).

**Fig. 4:**
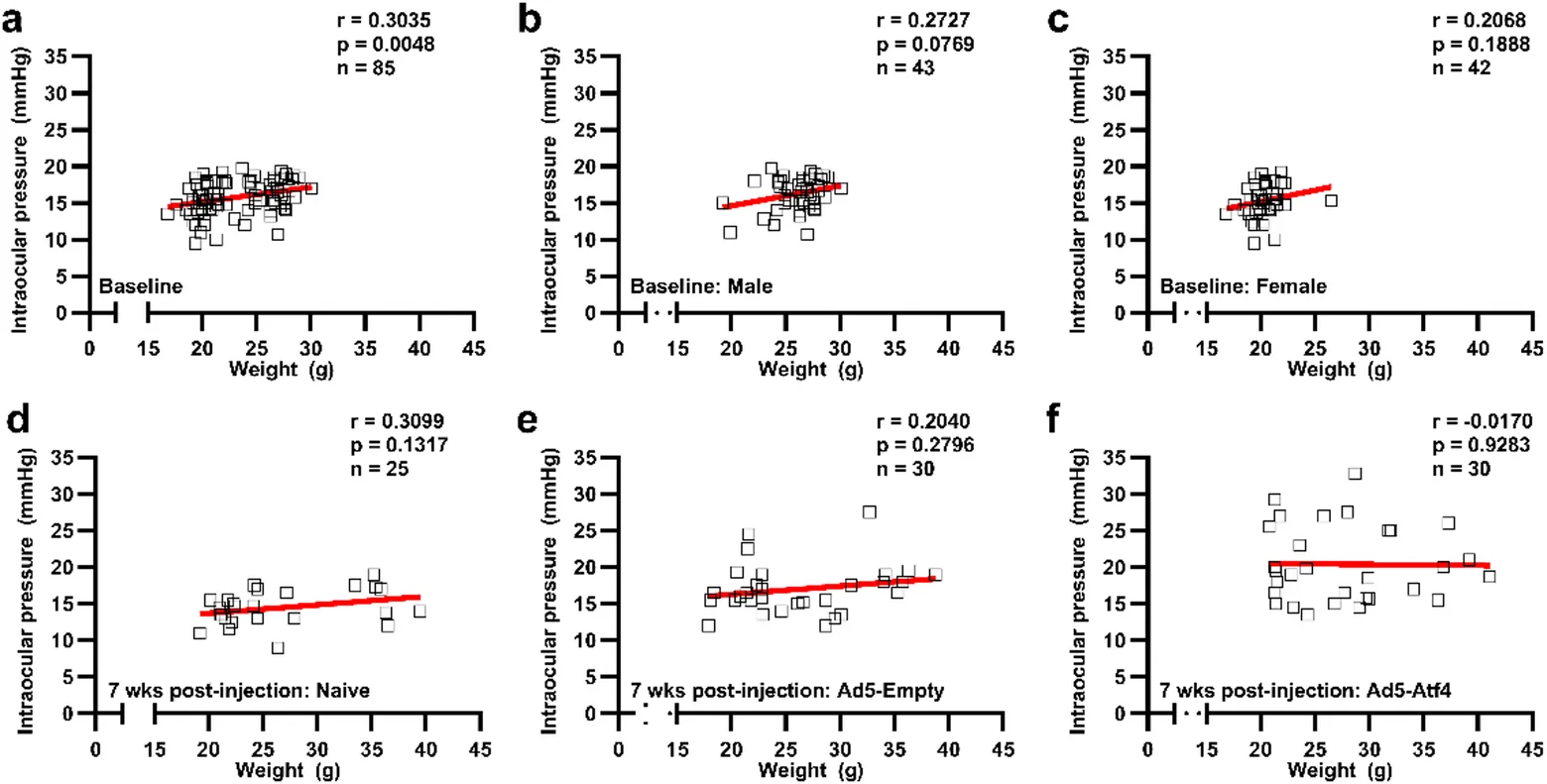
Correlation between IOP and body weight. (**a**) Baseline data from all mice of all cohorts in the study, replotted to show (**b**) only males and (**c**) only females. (**d–f**) 7-wks post-injection IOP for each ocular treatment group, including both NAM-diet and CON-diet. r = Pearson correlation coefficient, p = two-tailed p-value, n = number of mice (IOP values of both eyes were averaged to yield one IOP metric per animal).

A modest but significant baseline correlation between IOP and body weight was found (*r* = 0.304; *p* = 0.005; Pearson correlation analysis; Fig. 4A), suggestive in males (*r* = 0.273; *p* = 0.077; Fig. 4B) and not in females (*r* = 0.207; *p* = 0.189; Fig. 4C). The correlation subsequently weakened over time, with Naïve, Ad5-Empty, and Ad5-ATF4 treated groups having no significance (*p* = 0.132, *p* = 0.280, and *p* = 0.928, respectively; Fig. 4D and F), though the Naïve cohort maintained an r similar to the baseline cohort (*r* = 0.310).

### Influence of dietary NAM on the gut microbiome

**Fig. 5:**
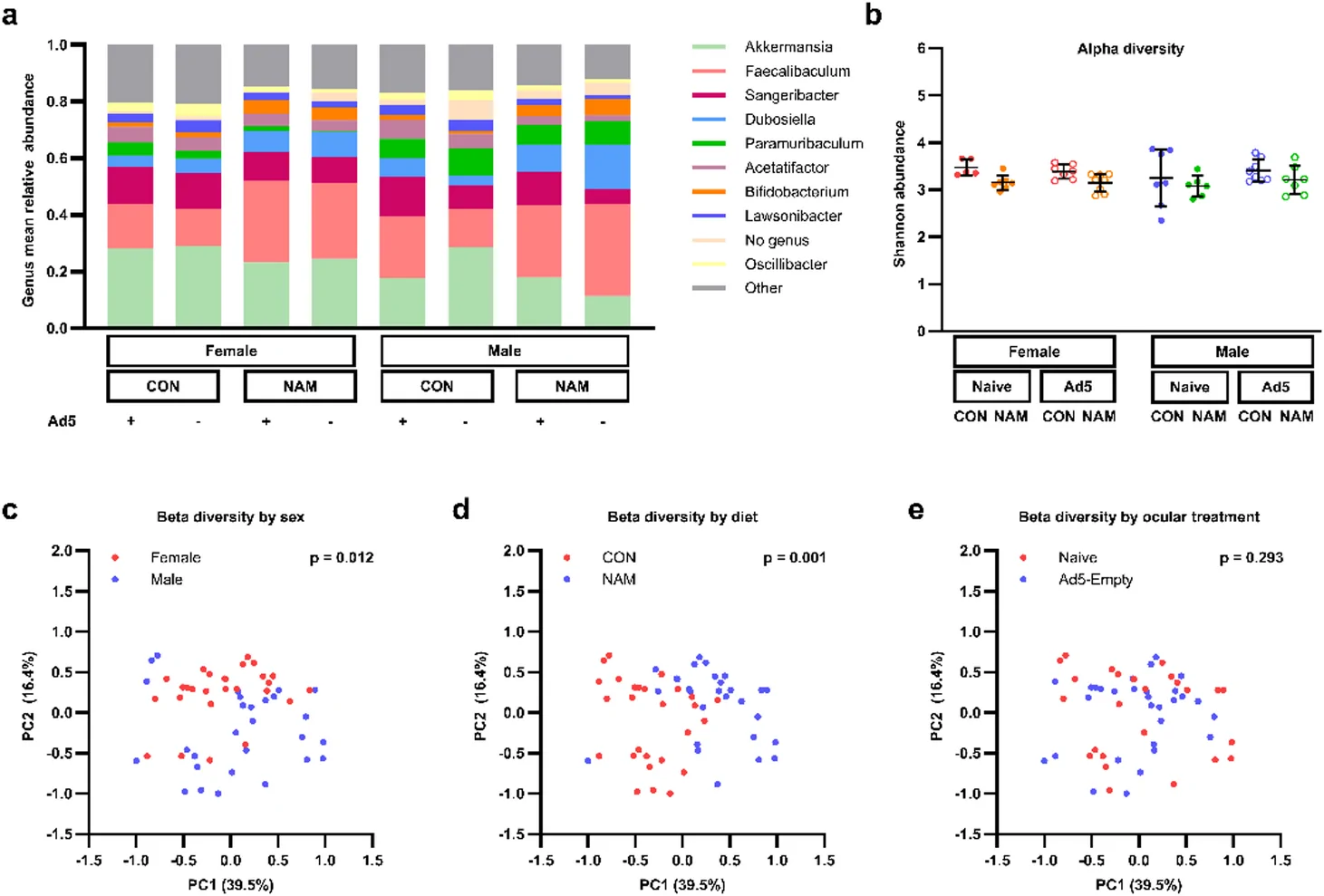
Effect of NAM on the gut microbiome. (**a**) Relative abundance of the ten most common genera. (**b**) Shannon alpha diversity. (**c–e**) Principal coordinates analysis plots with Bray-Curtis beta diversity by sex, diet, and ocular treatment. CON = CON-diet, NAM = NAM-diet, p = PERMANOVA.

In an exploratory gut microbiome analysis of Naïve and Ad5-Empty–injected mice, One-Codex-calculated relative abundance estimates were used to determine the number of taxa detected per sample, which was 93.4 ± 21.6 at the species level, and 34.3 ± 2.8 at the genus level (mean ± SD). There were multiple variations between groups at the genus level (Fig. 5A). The phyla *Verrucomicrobiota* (genus: *Akkermansia*), *Bacillota* (genera: *Faecalibaculum*, *Dubosiella*, *Acetatifactor*, *Lawsonibacter*,* Oscillibacter*), *Bacteroidota* (genera: *Sangeribacter*, *Paramuribaculum*), and *Actinobacteria* (genus: *Bifidobacterium*) dominated the microbiome composition in all cohorts. The NAM-diet was associated with multiple alterations in microbiome composition. Comparisons of the Shannon diversity index revealed an overall effect related to diet (*p* = 0.003, Mann-Whitey U test with Bonferroni correction for multiple comparisons), with the NAM-diet associated with lower microbiome alpha diversity (Fig. 5B). There were no individually significant differences between diets for each sub-group matched by sex and ocular treatment. Separation in beta diversity was observed (Fig. 5C, D and E) with differences based on sex (*p* = 0.012, PERMANOVA) and diet (*p* = 0.001), but not for ocular treatment (*p* = 0.293).

**Fig. 6:**
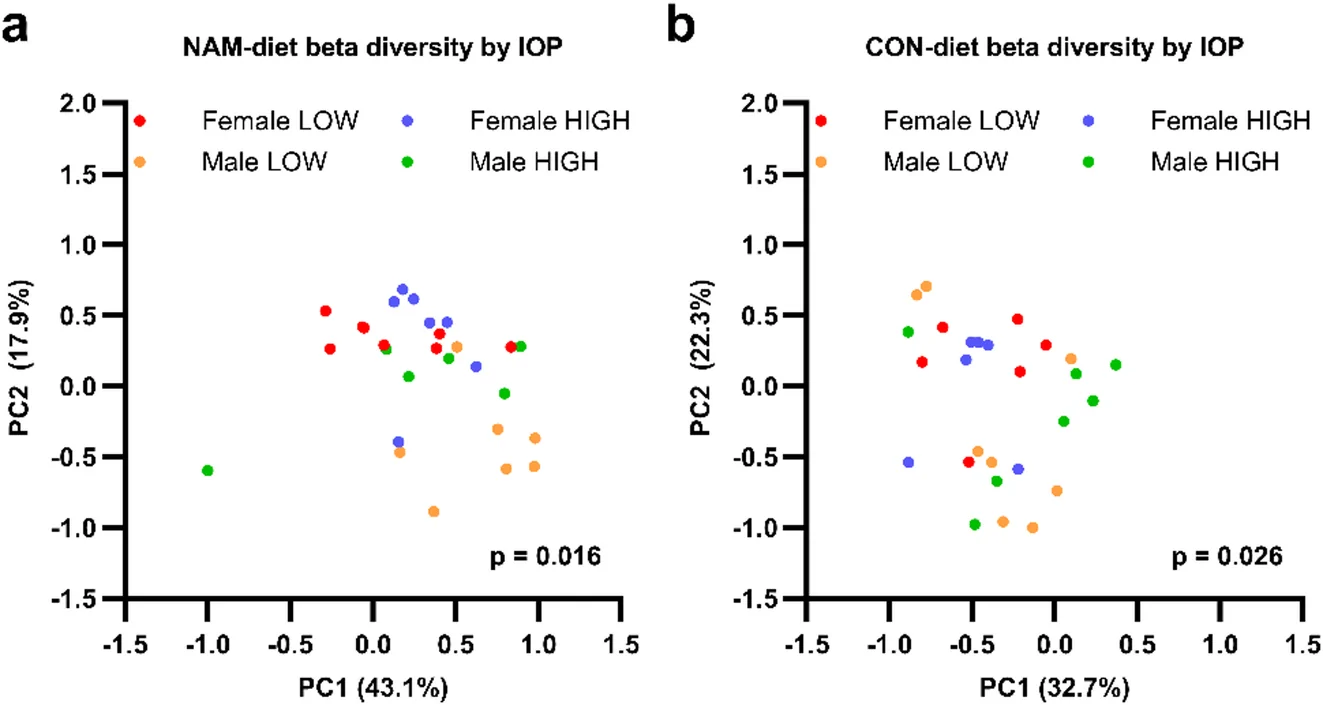
Associations between beta diversity and IOP. Principal coordinates analysis plots with Bray-Curtis beta diversity by max IOP for mice on (**a**) NAM-diet and (**b**) CON-diet. CON = CON-diet, NAM = NAM-diet, p = PERMANOVA.

**Fig. 7:**
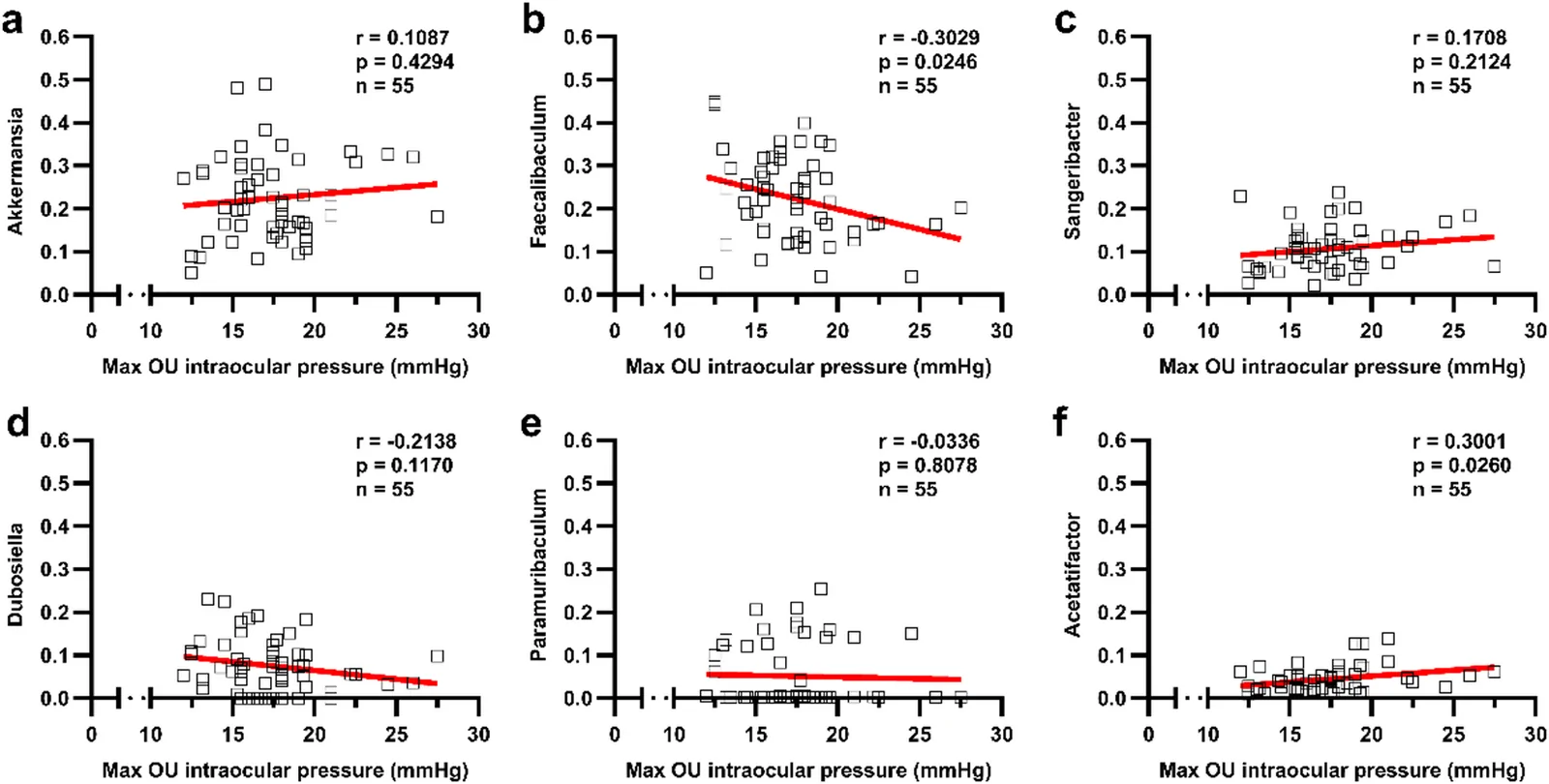
Associations between the gut microbiota and IOP. Correlations between relative abundance and max IOP. r = Pearson correlation coefficient, p = two-tailed p-value, n = number of mice (IOP values of both eyes were averaged to yield one IOP metric per animal), OU = both eyes.

We additionally tested groups of mice for possible associations between the microbiome and IOP. Because NAM-diet influences beta diversity and IOP, this analysis was done separately for mice in the NAM-diet and CON-diet groups (Fig. 6A and B). Mice from each group with microbiome data were divided according to max IOP, comparing beta diversity for half of the males and half of the females with the lower 50 percentiles of IOP for each sex to those with the upper 50 percentiles of IOP. This analysis detected a significant separation in beta diversity based on max IOP for both the NAM-diet (*p* = 0.016, PERMANOVA) and CON-diet groups (*p* = 0.026). In a second analysis, relative abundance for each of the six most abundant genera were tested for correlation to max IOP values for all mice with microbiome data (Fig. 7). Significant correlations were detected for two genera. Increased relative abundance of *Faecalibaculum* correlated with lower IOP (*r* = −0.303; *p* = 0.025, Pearson correlation; Fig. 7B) and increased relative abundance of *Acetatifactor* correlated with higher IOP (*r* = 0.300; *p* = 0.026; Fig. 7F).

## Discussion

Dietary supplementation with NAD^+ ^precursors such as NAM or NR protects retinal ganglion cells in glaucoma models, yet the extent to which dietary delivery produces non-neuronal changes relevant to glaucoma remains unclear^10–14,17,18^. The broad biological roles of NAD^+^, including regulation of mitochondrial function, redox homeostasis, and stress signaling, provide a conceptual framework capable of linking systemic metabolic effects to both neuronal and ocular pressure-related phenotypes^1,15–18^. In this study, we tested how a NAM-rich diet influenced IOP, body weight, and gut microbiome composition in healthy mice and in mice with Ad5-induced ocular hypertension.

The results showed that NAM-diet lowered IOP in a context-dependent fashion: it had no effect on the physiologically normal IOP of healthy mice but significantly reduced IOP in mice with Ad5-Empty–induced ocular hypertension. Ad5 is known to evoke an innate immune response when injected into the eye, readily apparent by slit-lamp as transient corneal clouding such as we observed here^20^. It is therefore important to note that the observed ocular hypertension in this study, and the NAM-associated effects on IOP, are occurring in the context of heightened ocular immune response. NAM-diet also influenced body weight and gut microbiome composition, indicating that NAD^+^ precursor-based therapies may offer multiple ocular benefits relevant to glaucoma, including neuroprotection and context-dependent reductions in pathologically elevated IOP. Our findings also identify two elements of general health—gut microbiome composition and body weight—that warrant further study.

Existing reports on the effects of NAM and NR on IOP have been mixed. Some animal-based studies observe a pro-hypertensive effect^11^; others report IOP lowering^13^, context-dependent effects^10,23^, or no influence at all^12,14,24^. Clinical trials of NAM as a potential neuroprotective agent for glaucoma patients have so far been unremarkable with respect to possible influences on IOP^25–28^. None of the published trials were designed with IOP as the primary outcome. Two studies observed no influence of NAM on IOP^27,28^, while one found a context-dependent reduction limited to normal tension glaucoma^26^. Our study found that dietary NAM reduced pathologically elevated IOP in a context-dependent manner. Prior studies show that systemic NAD^+^ precursors can influence intracellular NAD^+ ^levels in the aqueous humor outflow pathway of mice^13^. Functionally, NAD^+ ^precursor treatment is protective for oxidative stress and fibrosis in cultured human trabecular meshwork cell lines^29^, and mitigates abnormal extracellular matrix deposition and mitochondrial damage in a mouse model of glucocorticoid-induced glaucoma^13^. Like our findings with NAM, the study by Zhang et al. found that NR did not alter IOP in healthy C57BL/6J mice, only mice with induced pathology^13^. Additionally, the study by Tolman et al. recently found that NAM did not alter the IOP of healthy C57BL/6J mice, only in mice with *Lmxb1b*-mediated pathology^10,23^.

Our experimental design studied both Ad5-ATF4- and Ad5-Empty-treated cohorts―each yielding unexpected results. First, the IOP response of Ad5-ATF4 treated mice on CON-diet, though significantly different from Naïve mice, was muted compared to the existing published study^19^. Second, in partial contradiction to previous reports (which often used different strains, timeframes, and base diets)^19,30–33^, we observed increased IOP in the Ad5-Empty cohort. The inclusion of the Ad5-Empty group enabled separation of effects attributable to viral delivery and associated immune responses from those driven by ATF4 overexpression. Several studies have similarly utilized Ad5-Empty injections as a comparison for cargo carrying Ad5 vectors. Commonly, such studies do not statistically assess the impact of Ad5-Empty itself on IOP^19,34,35^. A few studies have reported that intravitreal injected Ad5-Empty had no statistically detectable influence on IOP of various cohorts (*n* = 6–8 mice per study; BALB/c and C57BL6-SV129 strains)^31–33^. In depth studies of intravitreal Ad5-Empty injections in larger cohorts of mice of additional strains have not been reported. However, it is known that intracameral or intravitreal injection of Ad5-Empty in mice can induce inflammatory responses^19,20^, which we speculate is the likely mechanism driving the elevated IOP detected in our study. Differences in mouse strain, age, diet, and statistical methodology likely all contribute to the variation present in the existing literature concerning effects of Ad5-Empty on IOP. Of note, we used a LMM to account for repeated measures and because it is robust to non-normal data. This approach contrasts the common practice of analyzing each time point independently with ANOVA. We also used a high-fat diet to match previous studies that is not used in routine husbandry. In our study, Ad5-Empty and Ad5-ATF4 groups exhibited statistically indistinguishable IOP elevations, suggesting that Ad5 injection itself can perturb ocular homeostasis. Interestingly, NAM-diet reduced IOP in an ATF4-sensitive manner: it lowered IOP in the Ad5-Empty treated cohort but not the Ad5-ATF4 treated cohort. In other tissues, ATF4 activation induces metabolic programs including NNMT, SLC7A11, and MTHFD2. These pathways regulate NAM metabolism and cellular redox pathways that could influence NAD^+ ^availability under stress conditions^36–38^. If a similar event occurred in trabecular meshwork cells, it could suggest a mechanistic basis for the attenuated response in Ad5-ATF4 treated mice.

As expected, high-fat diets and NAM supplementation both strongly influenced body weight^21,22^. High-fat diet is known to particularly cause weight gain in male mice^21^. Likewise, the influence of NAM-diet in our study was more notable in males, though also statistically significant in females. Some human studies suggest a correlation between body weight and IOP^39,40^, but the mechanisms remain unclear. We found a modest, significant baseline correlation between body weight and IOP that persisted over time in the Naïve cohort, but not in either Ad5-treated cohort. Thus, there were marked disassociations in responses to NAM over time. We speculate that the altered body weight to IOP correlations are driven by variable individual IOP responses to intravitreal Ad5 injections. Although all three cohorts showed similar body weight reductions, their IOP responses differed. This indicates that the observed NAM-induced IOP decrease in Ad5-Empty eyes is not secondary to changes in body weight.

NAM-diet also resulted in significant differences in the gut microbiome. Although not previously experimentally studied, such changes were theoretically predicted^41^. Dietary NR or NMN supplementation can modify mouse gut microbiome^42,43^, and gut microbiota can substantially modify NAD^+^ precursors^44,45^ or depend on NAD^+ ^precursors for growth^46^. We found that NAM-diet indeed altered gut microbial populations. NAM-diet in our study decreased alpha diversity, similar to findings in mice fed a NMN-diet^43 ^but not a NR-diet^42^. In humans, decreased alpha diversity is associated with several acute and chronic diseases^47^. We also found that beta diversity showed significant clustering of samples based on diet, consistent with findings from mice fed NMN-diet or NR-diet^42,43^. Most microbial changes were distinct from those found in analogous NR or NMN studies. This likely reflects differences in the NAD^+^ precursor used, as well as experimental variables such as base diet, sex, and age. Notably, microbial taxa showing exploratory associations with IOP in this study include the butyrate-producing genus *Faecalibaculum*^48,49^ and the acetate-producing genus *Acetatifactor*. Although neither genus has been directly associated with human glaucoma, both produce short-chain fatty acids (SCFAs), suggesting that shifts in microbial metabolic output may influence ocular physiology in a context-dependent manner. In human glaucoma cohorts, changes in butyrate- and acetate-producing taxa as a functional class have been reported^50–52^, supporting the interpretation that changes in microbial SCFA metabolism—rather than effects attributable to specific taxa—may be relevant to disease-associated ocular pathways. In light of the exploratory nature of our microbiome study, it is important to note that associations between microbiome composition and IOP do not establish causality and could alternatively result through mechanisms such as shared host physiological factors or reverse causation.

Several methodological considerations warrant mention. First, Ad5 injection disrupted the baseline correlation between left and right eye IOP, highlighting increased variability introduced by viral delivery, and potentially reflects differences in transduction efficiency or localized inflammatory responses. Second, IOP data from some of the sub-groups were not normally distributed, a statistical assumption some studies may be overlooking to their detriment. Third, our data reiterate a common finding―males and females differed in gut microbiome composition^53,54^. Experimentally, this difference may have broad relevance to other mouse studies based on dietary consumption of compound; ingested compounds may be differentially modified by the microbiome and contribute to sex-specific effects.

Caveats regarding study design include the exclusive use of C57BL/6J mice, which harbor a naturally occurring mutation in the nicotinamide nucleotide transhydrogenase gene (*Nnt*) which could influence relevant phenotypes^55,56^; and a NAM dosage exceeding that used in existing ophthalmic human trials^57^. The use of a high-fat base diet is an important experimental variable with the potential to confound interpretations because it independently influences both body weight and microbiome composition^58,59^. Even among mice, variability related to host genetic background, diet, housing environment, and gut region remains poorly understood, and it’s likely that all these factors are relevant and could give rise to context-specific results^53^. For example, mice in this study were not individually housed, so microbiome cross-transfer may have reduced inter-individual variability. Thus, findings may not generalize across diets, strains, or doses. Finally, there are multiple challenges related to species-specific differences that may influence the translational significance of our microbiome findings. These include differences in microbial metabolite sharing^46^, microbiome-mediated interconversion of NAD^+ ^precursors^60^,and gut anatomy^61^. These variables may alter how microbial abundance relates to disease relevance. In ongoing work, it would be worthwhile to study how NAM may influence aqueous humor dynamics, especially structure and function of a newly identified sub-population of trabecular meshwork cells recently implicated in an IOP-lowering effect of dietary NAM in *Lmx1b *mutant mice^23^.

In summary, NAM reduced ocular hypertension in a context-dependent fashion that was ATF4-sensitive. If these IOP-lowering effects extend to humans, NAM could serve as a multi-activity glaucoma therapy. The influence of NAM on alpha diversity and its possible effects on general health warrant additional study. NAM-induced changes in body weight may be beneficial for some individuals, such as overweight middle-aged adults, but detrimental for others, including older individuals for whom weight maintenance is challenging. Our study, using inbred mice with matched diets and environments, detected a correlation between NAM consumption and the microbial content and relative abundance in the gut microbiome. Analogous studies of NAD^+ ^precursors in humans have thus far been ambiguous^42,62^; it is unclear whether this reflects species-specific differences, necessarily limited experimental control, and/or limited statistical power^63^. Future studies incorporating absolute abundance may strengthen interpretation. The influence of NAM on microbial diversity and systemic physiology highlights the need for further investigation into how NAD^+^ precursors and microbial metabolites such as SCFAs interact and how these interactions may shape IOP regulation and glaucoma risk.

## Methods

### Mouse cohorts

Experiments were conducted in accordance with the recommendations of the Guide for the Care and Use of Laboratory Animals of the National Institutes of Health, approved by the Animal Care and Use Committees of the University of Iowa and adhered to the ARVO Statement for the Use of Animals in Ophthalmic and Vision Research. 8-week-old C57BL/6J mice were obtained from The Jackson Laboratory (Strain #: 000664) and acclimated to the University of Iowa vivarium while on a 4% fat NIH 31 diet. After 2 weeks, baseline IOP was recorded, and mice were split into 6 subgroups balanced by IOP and sex (i.e., mice were assigned subgroups to make the average baseline IOP of each subgroup approximately equal, with sexes split as equally as possible).

The subgroups differed by ocular treatment (both eyes: Naïve, Ad5-Empty injections, or Ad5-ATF4 injections), with approximately half of each subgroup subsequently receiving either the CON-diet or NAM-diet. The experimental design included an expectation that subgroups receiving intravitreal injections might experience greater attrition due to the added anesthesia procedures or complications related to intravitreal injections. To account for this, these subgroups were initially assigned extra mice. By design, no mice were excluded solely to balance subgroup sizes, which made slightly uneven group sizes likely (Supplemental Materials 2). To maintain housing density and minimize fighting, mice were housed with two mice per pen (or one mouse per pen if the group size was uneven).

Prior to initiation of the study, one mouse was found to have low body weight secondary to malocclusion and was excluded from data measurements but retained to maintain housing density. No other mice were excluded, and no mice were lost due to attrition or experimental manipulations during the experimental time course. Sample sizes were based on prior studies that detected significant differences in IOP in response to Ad5-ATF4^19^. The final number of mice analyzed in each subgroup were: Naïve CON-diet *n* = 12 mice (5 female, 7 male), Naïve NAM-diet *n* = 13 mice (7 female, 6 male), Ad5-Empty CON-diet *n* = 15 mice (7 female, 8 male), Ad5-Empty NAM-diet *n* = 15 mice (8 female, 7 male), Ad5-ATF4 CON-diet *n* = 14 (7 female, 7 male), Ad5-ATF4 NAM-diet *n* = 16 mice (8 female, 8 male). Mice within each pen received the same diet but did not necessarily have the same ocular treatments. To maintain consistent lighting conditions, pens from different groups were interspersed within the housing rack to avoid potential environmental biases. All pens contained dry bedding (Cellu-dri; Shepherd Specialty Papers, Kalamazoo, MI, USA) and were kept in a controlled 21°C environment with a 12-hour light/12-hour dark cycle.

### Experimental diets

The base chow for both diets was TestDiet 5W80 (TestDiet, Richmond, Indiana), which was modified by the manufacturer to contain 10,000 PPM nicotinamide (Niacinamide-USP, catalog number NI105, Spectrum Chemical Mfg Corp, New Brunswick, New Jersey) for the NAM-diet. All mice received filtered water (Filter Bank Station, Avidity Science, Waterford, WI) ad libitum, replaced weekly, with either no supplements or supplemented with 6 g/L nicotinamide. The combined NAM dosage from food and water was 2,000 mg/kg body weight/day, matching the “NAM^Hi^” diet previously utilized by Williams et al.^10^

### Adenoviral injection

Matching the prior study by Kasetti et al.^19^, Ad5-ATF4 and Ad5-Empty virus preparations were commercially obtained from ViraQuest (North Liberty, Iowa, USA). Ad5-ATF4 was originally generated and described by Dr. Rajiv Ratan^64^. Mice were anesthetized with a mixture of ketamine/xylazine (intraperitoneal injection of 87.5 mg ketamine + 12.5 mg xylazine/kg body weight; ketamine and xylazine from Dechra Veterinary Products, Overland Park, KS, USA). In all mice, whiskers and eyelashes were trimmed, eyes were gently flushed with saline, and 0.5% proparacaine hydrochloride ophthalmic solution (Bausch + Lomb, Bridgewater, NJ, USA) was applied for local ocular anesthesia. Using a 32-gauge needle on a Hamilton syringe under a microscope, mice in the Ad5-treated subgroups received a single bilateral intravitreal 1-µl injection containing 2 × 10^7^PFU per eye of Ad5-ATF4 or Ad5-Empty virus with 0.1% fluorescein (Akorn, Buffalo Grove, IL, ISA) to visualize successful administration. This design was selected to match that of Kasetti et al. as closely as possible^19^. During anesthesia recovery, Optixcare Eye Lube (CLC MEDICA, Ontario, Canada) was applied to maintain corneal hydration and mice were provided supplemental indirect warmth. 20% of the cohort began treatment each day over 5 days to streamline workflow. Subgroups were intermixed within each daily batch to evenly distribute treatment conditions throughout the week. The day of treatment determined the schedule for subsequent IOP measurements and weight assessments. To standardize treatment, Naïve controls were anesthetized and handled identically to other treatment groups. Following anesthesia recovery, fresh cages and diets were provided; after 5 days in BSL2C, mice were returned to standard housing.

### Measurement of IOP

IOP measurements were taken every other week, alternating weeks with facility cage changes (to minimize possible stressors that could confound results), by a masked observer throughout the study (Supplemental Materials 1). Because subgroups entered the 7-week study in a stepwise fashion, only 20% of mice in the study were measured on each recording day, allowing all IOP measurements to be collected within a consistent 3-hour window (7.5–10.5 h after start of the lights on period). IOP of both eyes was measured using the TonoLab rebound tonometer (iCare Finland Oy, Helsinki, Finland). Mice were lightly sedated with 2% isoflurane at a flow rate of 800 mL/min (SomnoSuite, Kent Scientific, Torrington, CT, USA). While isoflurane anesthesia can influence IOP, all measurements were performed under standardized conditions. Each mouse was placed in an induction chamber for 3 min, transferred to a nose cone, had whiskers trimmed, and had five completed measurements per eye (each based on 6 rebounds). The three complete measurements closest to the median were averaged to obtain a final value. The total sedation time did not exceed 5 min. No IOP data were excluded from the study.

### Slit-lamp examination

Anterior chamber phenotypes were assessed in conscious mice by an observer masked to treatment, using a slit-lamp at 25X magnification (SL-D7; Topcon, Tokyo, Japan), and photo-documented using a digital camera (D800; Nikon, Tokyo, Japan). All photographs were captured with identical settings and image processing. For every mouse, both eyes were examined by broad beam illumination at 1-, 3-, and 7-weeks of the study, 1–3 days following IOP recordings. Only mice with overt injection complications, such as blood in the anterior chamber, would have been excluded from the study based on appearance (no eyes met this criterion; all eyes were included).

### Body weight and microbiome analyses

Body weight was measured at baseline, 5-, and 7-weeks post-injection (Supplemental Materials 1). Two fecal pellets were collected from individual Naïve and Ad5-empty mice 6 weeks after initiating the high-fat diets into tubes of solution provided by Transnetyx (Cordova, TN, USA). Week 6 was selected to capture microbiome composition following sustained dietary exposure while minimizing potential confounding to terminal time points. The Naïve cohort was used to assess the effect of NAM on the gut microbiome in mice with physiologically normal IOP. The Ad5-Empty cohort was used to assess the gut microbiome in a pathologic context where NAM elicited a decreased IOP. The gut microbiome of the Ad5-ATF4 cohort was not assessed because NAM had no effect on pathologic IOP in this context.

Samples were sent to Transnetyx for shallow shotgun whole-genome sequencing, with a read depth of 2 million paired-end reads. Sequence data from Transnetyx were automatically uploaded to One Codex and aligned to the One Codex reference database via *k*-mer based classification where *k* = 31, with exclusion of mouse host DNA and other non-microbial sequences. Sequencing and reference genome artifacts were filtered based on the relative frequency of unique *k*-mers. The One Codex platform was used for most analyses^65^, including: (1) “relative abundance” at the genus level: an estimate of abundance based on the depth and coverage across every available reference genome by One Codex which takes into account genome sizes of the microbiota and the number of *k*-mers that uniquely identify a species. (2) “Alpha diversity”: a measure of “with-in” diversity of a sample. The Shannon Index is one metric for alpha diversity which estimates both species richness and evenness, with weight on the richness. (3) “Beta diversity”: a measure of differences between samples. Principal Coordinates Analysis (PCoA) is an approach for plotting beta diversity which essentially treats each species as a single distinct dimension in pair-wise comparisons and then uses dimension reduction techniques to arrange the data within a lower-dimensional space―maximizing the proximity of similar samples and distancing those that are dissimilar.

### Statistical analysis

The primary outcome measure was intraocular pressure (IOP). Body weight and gut microbiome composition were evaluated as secondary exploratory endpoints.

#### For the study of IOP:

Baseline IOP was used to match groups to promote a simplified interpretation of the resultant graphs. IOP was averaged from the left and right eye measurements per mouse per time point. Because the experiment included repeat measures over time, we used a LMM for statistical testing^66^ for its robustness in analyzing complex study designs. Models were visually assessed using residual and fitted value diagnostics to evaluate homoscedasticity and linearity. Normality of residuals was examined using Q-Q plots rather than relying on assumptions of normally distributed raw IOP values, as the LMM framework is robust to moderate deviations from normality in outcome distributions. In addition, we note that some IOP data were not normally distributed, which motivated our use of LMMs rather than timepoint-specific ANOVA. Using R, the LMM was fitted for the entire data set with fixed effects of sex, time, injection, and diet using the lmer function in the lme4 package (version 1.1.37). No interaction effects with sex were evaluated. Post-hoc tests, which were limited to the 3-, 5-, and 7-weeks post-injection time points, used pairwise comparisons of the EMMs with Tukey adjustment for multiple comparisons. The R package emmeans (version 2.0.0) was used for these post-hoc tests.

#### For the study of body weight:

The effect of diet on body weight was analyzed in the same way as in the study of IOP, except that the post-hoc tests were conducted at the 5- and 7-week post-injection time points.

#### For microbiome studies:

Relative abundance, alpha diversity, and beta diversity were all calculated using online analysis tools of One Codex. Non-parametric statistical tests were used to assess differences in alpha diversity between cohorts, using the Mann-Whitney U test with Bonferroni correction for multiple comparisons to assess the effect of a single variable (i.e., overall effect of sex, diet, or ocular treatment) and the Kruskal-Wallace test to assess differences between more than two groups. The significance for beta diversity was calculated by PERMANOVA. Linear regression and Pearson correlation analyses were performed with GraphPad Prism 10.5.0 for Windows (GraphPad Software, Boston, Massachusetts, USA) and p values were not corrected for multiple comparisons. For analyses involving “max IOP”, the average IOP from the left and right eye measurements per mouse per time point were used, with the max value from weeks 3–7 defining the max. Statistical p values < 0.05 were considered significant, 0.05–0.10 suggestive, and > 0.10 not significant. While both sexes were represented in each experimental group, sample sizes were optimized for detecting treatment effects on IOP rather than sex-specific differences, limiting statistical power for formal sex-stratified analyses.

## Supplementary Information

Below is the link to the electronic supplementary material.

Supplementary Material 1

Supplementary Material 2

## Data Availability

The IOP and body weight datasets are included as supplemental materials. Microbiome data are publicly available on NCBI BioProject accession: PRJNA1451933. Interactive data are available through One Codex, project aba007c6dc4e4813, at https://app.onecodex.com/projects/aba007c6dc4e4813.
