# Supplementary material for: Systemic influences of dietary NAD+ supplementation relevant to the search for ocular hypertensive therapies for glaucoma
Source: Sci Rep. 2026 Jul 29;16:34891. doi: 10.1038/s41598-026-58123-8 (PMC13713582; doi:10.1038/s41598-026-58123-8)
Supplement: Supplementary file 1 [file 41598_2026_58123_MOESM2_ESM.docx]

**Supplemental Materials**

**Supplemental Figure 1. IOP correlation between right and left eyes measured by rebound tonometry.** **(A)** Baseline IOP from all mice of all cohorts in the study. **(B)** 7-wks post-injection IOP for Naïve group, including both NAM- and CON- diets. **(C)** 7-wks post-injection IOP for Ad5-Empty-injected pairs of eyes, including both NAM- and CON- diets. **(D)** 7-wks post-injection IOP for Ad5-ATF4-injected pairs of eyes, including both NAM- and CON- diets. Each point represents the pair of eyes from one mouse.

**Supplemental Figure 2. Flowchart of mouse allocation and retention.**
